# Suspected Achilles Tendon Ruptures and the Course of Action: Assessing Compliance With Local Protocols

**DOI:** 10.7759/cureus.95225

**Published:** 2025-10-23

**Authors:** Hassan Imtiaz, Ayesha Naz, Thet Paing Oo, Atizaz A Jan, Talha Ahmed, Omran Alkhatib, Nasir Nasim

**Affiliations:** 1 Trauma and Orthopaedics, University Hospitals Dorset, Poole, GBR; 2 Trauma and Orthopaedics, Poole Hospital, University Hospitals Dorset, Poole, GBR

**Keywords:** achilles tendon injury, ankle and foot, msk radiology, quality improvement (qi), ultrasound (u/s)

## Abstract

Introduction: Achilles tendon rupture is a significant injury that can cause prolonged disability and impact quality of life. It typically occurs during sports or activities involving sudden loading of the tendon, most often in active adults. Early recognition and appropriate management are vital to reduce complications, optimise healing, and support timely return to function. Clinical pathways provide structured guidance for consistent care, including immobilisation, prophylaxis, imaging, and specialist follow-up. Variation in adherence to such pathways may compromise outcomes. Clinical audit provides a means to evaluate current practice against pathway standards, while closed-loop audits allow assessment of targeted interventions aimed at improving compliance.

Methods: A closed-loop audit was performed at a large district general hospital to assess compliance with a locally developed Achilles tendon rupture pathway. The first cycle reviewed 138 patients between October 2022 and September 2023. Following the dissemination of results, display of posters in relevant areas, and introduction of a dedicated “Ultrasound Achilles” request form, a second cycle reviewed 44 patients between March and June 2024. Standards included venous thromboembolism (VTE) prophylaxis, weight-bearing advice, immobilisation, ultrasound imaging within 14 days, and referral to a virtual fracture clinic (VFC). Data were analysed using chi-squared tests.

Results: In cycle 1 (*n* = 138), compliance was as follows: VTE prophylaxis 110/138 (80%); immobilisation in boot 110/138 (80%); ultrasound within 14 days 59/138 (43%); non-weight-bearing advice 91/138 (66%); and VFC referral 132/138 (96%). In cycle 2 (*n* = 44), after interventions including pathway dissemination and the introduction of a dedicated “Ultrasound Achilles” request form, compliance improved in several domains: VTE prophylaxis 35/44 (80.5%, *p* = 0.91); immobilisation 38/44 (86%, *p* = 0.04); ultrasound within 14 days 26/44 (59%, *p* = 0.03); non-weight-bearing advice 29/44 (66%, *p* = 0.99); and VFC referral 44/44 (100%, *p* = 0.01). No re-ruptures or venous thromboembolic events were recorded in either cycle.

Conclusion: Targeted interventions improved compliance with several aspects of the pathway, though VTE prophylaxis prescribing showed little progress. Ongoing education, structured pathways, and regular re-audits are essential to sustain improvement and ensure optimal outcomes.

## Introduction

Achilles tendon rupture is one of the most common tendon injuries in adults, frequently occurring in middle-aged individuals during recreational sports or sudden loading activities [1,2]. Prompt diagnosis and evidence-based management are essential to minimise long-term morbidity, which may include persistent weakness, gait disturbance, or re-rupture [3].

Clinical management strategies have evolved substantially, with both surgical and non-surgical approaches demonstrating favourable outcomes when undertaken within structured treatment pathways [4,5]. National and international guidelines recommend key elements in the acute management of suspected Achilles tendon rupture. These include venous thromboembolism (VTE) risk assessment and prophylaxis, appropriate immobilisation in a functional boot with wedges, timely ultrasound imaging, and referral to specialist clinics for follow-up [6-9].

In February 2021, University Hospitals Dorset (UHD) introduced a clinical pathway for suspected Achilles tendon ruptures to standardise care across emergency, orthopaedic, and fracture clinic services. This pathway recommended that patients presenting in the emergency department with suspected Achilles tendon rupture should be given a boot with wedges to support the foot, advised to be non-weight bearing on the affected limb, and be prescribed appropriate VTE prophylaxis for the duration of non-weight bearing. Also, they should have an outpatient ultrasound requested to confirm the Achilles rupture, along with a referral to the virtual fracture clinic, with the primary objective being to have the scan prior to being seen in the face-to-face fracture clinic (booked following review in the virtual fracture clinic). This would allow the clinician during the face-to-face clinic to discuss whether surgery is indicated or not, given that the ultrasound scan has been performed by that time.

Despite implementation, variation in adherence is common when new pathways are introduced, particularly across multiple clinical teams. Regular clinical audit provides an essential tool to evaluate compliance, identify gaps, and inform targeted quality improvement interventions [10-12].

This study presents the results of a closed-loop two-cycle clinical audit evaluating compliance with the UHD Achilles tendon rupture pathway and the impact of pathway-based interventions. By comparing practice across two audit cycles, the study aimed to assess the effectiveness of implemented interventions and to guide further service improvements.

## Materials and methods

This closed-loop clinical audit was performed at University Hospitals Dorset. Data was collected retrospectively for both audit cycles. Adult patients (age > 18 years) presenting with suspected Achilles tendon rupture were included across two audit cycles. These were identified from emergency department daily logs of patients, for both minor and major units. Patients with open wounds or lacerations over the Achilles tendon, those with incomplete records, or those who required an inpatient stay at initial presentation were excluded (as they would not fulfil the criteria for referral to VFC or booking for an outpatient ultrasound scan). Data sources included Agyle Health (an electronic patient record system that analyses patient data in real time) for emergency department logs, Picture Archiving and Communication System (PACS) for ultrasound studies, and Electronic Patient Records (EPR) to assess virtual fracture clinic review notes. 

The first audit cycle collected retrospective data from October 2022 to September 2023, while cycle 2 followed the implementation of interventions and included patients between March and June 2024.

Audit standards were derived from the UHD Achilles tendon rupture pathway, with compliance targets set at 100%. The standards included VTE prophylaxis prescribed, appropriate immobilisation in a functional boot with wedges, ultrasound performed within 14 days of injury, clear documentation of weight-bearing advice, and referral to a virtual fracture clinic (VFC). Target compliance was set at 100% following agreement amongst the orthopaedic and emergency department teams. These standards are presented in Table 1. 

**Table 1:** Audit standards VTE: venous thromboembolism

Standard	Target Compliance (%)
VTE prophylaxis prescribed	100%
Boot with wedges applied	100%
Ultrasound within 14 days	100%
Non-weight-bearing advice	100%
Virtual fracture clinic referral	100%

Interventions between cycles included the dissemination of results via email, along with recommendations and improved accessibility of the pathway by means of displaying it in relevant clinical areas (emergency department and orthopaedic team room, fracture clinic). Furthermore, to improve the timing of performing ultrasound within 14 days of initial presentation, an additional imaging request form called “Ultrasound Achilles” was introduced in addition to the existing “Ultrasound Ankle”. The aim of this intervention was to alert the radiographers to the nature of the request so that they could comply with the 14-day target set out in the suspected Achilles rupture pathway.

Data was collected and statistical analysis performed using Microsoft Excel. Chi-squared tests were applied to compare domain-specific compliance proportions between the two audit cycles. A p-value of <0.05 was considered statistically significant. This audit was registered with the local trust clinical governance department and did not require ethical approval for the institutional review board. 

## Results

A total of 138 patients were included in cycle 1, with 44 patients in cycle 2. During the first cycle, 110 patients (80%) were prescribed VTE prophylaxis and immobilised in a boot with wedges, with 132 (96%) referred to VFC. Only 91 (66%) patients were given non-weight-bearing advice, with only 59 (43%) of the patients having their ultrasound scans within the 14-day window. Following the implementation of targeted interventions, the second audit cycle was conducted. VTE prophylaxis was prescribed in 35 patients, showing a modest improvement from 80% (previous cycle) to 80.5%. Immobilisation in a boot was seen in 38/44 patients, reflecting an improvement to 86%. 26/44 (59%) patients got their ultrasounds done within 14 days, reflecting the area of highest improvement, going up from 43% to 59%. Compliance was similar to the previous audit cycle for the remaining parameters; 29/44 (66%) patients were advised on non-weight bearing, and all 44 patients (100%) were referred to VFC. The distribution of compliance across audit standards is shown in Table 2.

**Table 2:** Comparison between audit cycles * Change of percentage compliance between audit cycles, with respect to cycle 2 VTE: venous thromboembolism

Standard	Cycle 1 (n,%)	Cycle 2 (n,%)	Change* (%)
VTE prophylaxis	110 (80%)	35 (80.5%)	+ 0.5%
Boot immobilisation	110 (80%)	38 (86%)	+ 6%
Ultrasound <14 days	59 (43%)	26 (59%)	+ 16%
Non-weight-bearing advice	91 (66%)	29 (66%)	0%
Virtual Fracture Clinic referral	132 (96%)	44 (100%)	+ 4%

Statistical testing demonstrated significant improvements in immobilisation (χ² = 4.13, p = 0.04), ultrasound within 14 days (p = 0.03), and VFC referral (p = 0.01). VTE prophylaxis prescribing did not significantly change (p = 0.91), while documentation of weight-bearing advice showed no improvement (p = 0.99). No re-ruptures or VTE events were recorded during either audit cycle. Statistical analysis is summarised in Table 3.

**Table 3:** Statistical analysis * Chi-squared value, ** p-value of < 0.05 is considered statistically significant VTE: venous thromboembolism

Standard	χ² value*	p-value**	Statistically Significant (Yes/No)
VTE prophylaxis	0.01	0.91	No
Boot immobilisation	4.13	0.04	Yes
Ultrasound <14 days	4.64	0.03	Yes
Non-weight-bearing advice	0.00	0.99	No
Virtual Fracture Clinic referral	6.12	0.01	Yes

## Discussion

This closed-loop audit demonstrated measurable improvements in compliance with a locally developed Achilles tendon rupture pathway following targeted interventions. Notable progress was achieved in appropriate immobilisation, timely ultrasound assessment, and referral to the virtual fracture clinic. However, compliance with VTE prophylaxis prescribing and provision of consistent weight-bearing advice remained unchanged, highlighting ongoing gaps in pathway implementation.

The clinical significance of these improvements is supported by a growing body of literature linking pathway adherence with enhanced patient outcomes. The observed increase in early functional immobilisation aligns with evidence demonstrating that rehabilitation in a controlled ankle motion (CAM) boot with heel wedges promotes faster recovery and lower re-rupture rates compared to traditional casting approaches [13,14]. This is consistent with the accelerated rehabilitation protocols described by Brumann et al. [13] and Olsson et al. [14], both of whom reported improved functional outcomes without increased complication rates. By embedding such principles into the local pathway, this audit mirrors international trends toward early mobilisation and standardised conservative management, as recommended in the AAOS and BOA guidelines [6,7].

Timely ultrasound assessment also showed significant improvement, which is consistent with findings from previous studies emphasising the importance of early imaging in confirming rupture, identifying partial injuries, and guiding management [15,16]. Improved referral rates to the virtual fracture clinic reflect growing evidence supporting specialist oversight in tendon rupture management. Fracture clinics facilitate multidisciplinary decision-making, continuity of care, and standardisation of advice, all shown to reduce treatment variability and improve adherence to evidence-based protocols [17,18]. Comparable initiatives, such as the virtual fracture clinic models described by British Orthopaedic Association (BOA) and Royal College of Surgeons (RCS) England, have demonstrated similar benefits in both efficiency and patient outcomes [8]. Nilsson-Helander et al. [19] highlighted that accurate early diagnosis is crucial for selecting appropriate non-operative pathways and avoiding unnecessary surgical intervention. Thus, enhanced imaging compliance in this audit may have contributed to the absence of re-ruptures across both audit cycles.

In contrast, the lack of improvement in VTE prophylaxis prescribing remains a key concern. Achilles tendon rupture is associated with a substantially elevated risk of deep vein thrombosis, largely due to immobilisation and reduced calf muscle activity [18,19]. Svedman et al. [18] identified a correlation between DVT and poorer patient-reported outcomes, reinforcing the need for prophylactic vigilance. Despite the National Institute for Health and Care Excellence (NICE) guidance (NG89) advocating individualised risk assessment [9], under-prescription remains a common challenge in musculoskeletal injuries. Studies suggest barriers may include clinician uncertainty about indications, inconsistent documentation, or patient reluctance [9,18]. Embedding automated prompts within electronic prescribing systems, as supported by previous quality improvement initiatives, may help address these gaps.

Similarly, continued variability in weight-bearing advice reflects the wider heterogeneity observed in clinical practice. While some clinicians remain cautious, evidence increasingly supports early weight-bearing under protection. Costa et al. [20] and Aufwerber et al. [21] both reported superior functional recovery with immediate or early mobilisation compared to delayed protocols, without increased risk of re-rupture. These findings underpin modern functional rehabilitation strategies, yet translating them into consistent bedside practice remains challenging. The persistent inconsistency observed here underscores the importance of reinforcing guidance through education, multidisciplinary agreement, and clear protocol documentation.

The absence of re-ruptures in either audit cycle aligns with the low complication rates reported in recent meta-analyses comparing operative and non-operative care [10,19,22]. Contemporary studies such as those by Willits et al. [5] and Ochen et al. [10] demonstrate equivalent functional outcomes and re-rupture rates when functional bracing protocols are followed, supporting the efficacy of structured conservative management within a clear pathway framework.

Finally, this audit highlights the broader value of closed-loop auditing as a quality improvement tool. Repeated audit cycles, combined with targeted education and feedback, have been shown to drive sustained adherence to best practice [23]. In this context, local audits not only ensure alignment with national standards but also identify process-level issues, such as documentation gaps or variation in advice that are not captured through outcome metrics alone.

This single-centre, retrospective audit is limited by potential documentation bias, as some aspects of care, such as verbal advice or prophylaxis counselling, may not have been consistently recorded. The smaller sample size in the second cycle reduced statistical power and may have limited the detection of subtle improvements. As a process-focused audit, patient-centred outcomes such as functional recovery, satisfaction, or complications were not evaluated. Additionally, external factors such as staff turnover or service pressures between audit cycles could have influenced compliance independent of the interventions. Despite these constraints, the study provides a valuable reflection of real-world practice and demonstrates how closed-loop auditing can drive measurable quality improvement.

## Conclusions

Implementation of a standardised Achilles tendon rupture pathway led to improvements in several aspects of care, particularly immobilisation, ultrasound timing, and referral to specialist clinics. However, prescribing of VTE prophylaxis and provision of weight-bearing advice remain areas for improvement. Sustained progress will depend on ongoing education, embedding pathway prompts in clinical systems, and further closed-loop audits.
